# For Those with Inadequate Legal Documents, COVID-19 Vaccination Remains Challenging

**DOI:** 10.33552/WJGWH.2021.05.000609

**Published:** 2021-09-07

**Authors:** Katherine Jennifer Kramer, Capricia Bell, Maurice-Andre Recanati

**Affiliations:** 1 Department of Obstetrics and Gynecology, St. Vincent’s Medical Centers Manhattan, USA; 2 Medical Student, Wayne State School of Medicine, USA; 3 Department of Obstetrics and Gynecology, Wayne State University School of Medicine, USA; 4 NIH-Women’s Reproductive Health Research (WRHR) Scholar, Department of Obstetrics and Gynecology, Wayne State University, USA

**Keywords:** Undocumented, Vaccination, COVID-19, Identification, Vulnerable

## Abstract

Mass vaccination against COVID-19 is ever urgent as the incidence of infection with the more contagious and severe Delta variant continues to rise. Though the COVID-19 vaccination is recommended for eligible individuals over the age of twelve and has become widely available to all, it remains elusive for poorly document individuals.

## Opinion

Her name was Maria. She was employed by my next-door neighbor as a house cleaner and was an undocumented alien living in fear of being discovered and deported. Several days ago, my neighbor’s daughter knocked on my door frantically. She explained that Maria had collapsed while cleaning the bathroom and felt extremely short of breath. My first thought was that she had contracted COVID-19 as I raced down the hallway to evaluate her. As I spoke with her in Spanish and examined her, it became clear that she was very anemic as she had been bleeding nonstop for several weeks. She refused to be taken to a hospital or see a doctor as she had neither insurance nor valid legal documentation. So, as a board-certified OB/GYN and a decent human being, I decided to treat her myself at no cost. Quickly, I discovered the limitations that exist in the health care system for those that are undocumented. Simple tasks such as ordering a complete blood count or medications become nearly impossible tasks.

One of my goals was to get Maria vaccinated for COVID-19 since she has a high risk of becoming severely ill from a COVID-19 infection due to her multiple medical conditions [1]. Additionally, ethnic minorities and socially vulnerable individuals are more likely to experience an increase in disease severity related to COVID-19 resulting in hospitalization, critical care, mechanical ventilation, and even death [2]. The Centers for Disease Control and Prevention (CDC) recommends anyone age twelve years and older to plan to get the COVID-19 vaccine [3] and while both the Department of Homeland Security (DHS) and the CDC promised to make vaccines universally available to those uninsured and those undocumented [4,5] there are significant obstacles that prevent this reality. In the state of New York, proof of age using a valid picture identification is required for obtaining a vaccine [6] which became clear to me after multiple telephone calls to hospitals and city and state health department officials. Maria didn’t have legal identification papers (or at least any that she cared to share with me) and, as such, was turned away from a mass vaccination site and told that she was ineligible for receiving the COVID-19 vaccine.

Over ten million undocumented individuals live in the United States each year [7] and without adequate identification and access to vaccination, millions of individuals are highly vulnerable to contracting and inevitably spreading COVID-19. These individuals are undoubtedly the type of patients that need to be vaccinated from a public health stance if not only for their safety but also for the safety of others. In Maria’s case, should she become infected, she could spread the virus to many others as she lives in close quarters with her family and other undocumented individuals. Her social and health circumstances increase her vulnerability, and she could potentially end up in an intensive care unit on a ventilator, costing taxpayers hundreds of thousands of dollars [8]. Increasing COVID-19 hospitalizations have a negative net financial impact on health care costs averaging 50 billion dollars monthly according to the American Hospital Association [9]. These rippling effects could have far-reaching financial and public health consequences.

The COVID-19 vaccine, particularly any RNA-based vaccine, has an excellent safety profile, and indications for vaccinations generally far outweigh vaccine-related risks [10]. We urge the federal government, state and local health departments, and hospitals and clinics to make the COVID-19 vaccine available without the need to show identification or prove state residency for adults. In many other cases, such as emergency contraception or IV needle exchange, the United States has recognized harm reduction programs as integral in attenuating public health consequences [11,12]. Potentially, being able to access vaccines without any barriers would reduce the large number of unvaccinated individuals who are holding out on receiving the vaccine due to lack of legal documentation. This includes not only ten million [7] undocumented immigrants but also the two million individuals who have warrants out for their arrest [13], nine million LGBTQI individuals [13], specifically trans and non-binary individuals who may have identification documents that do not match who they are, and those that simply fear being cataloged into government vaccine databases.

While one reason for verifying a patient’s identity is to properly document that an individual has been vaccinated, a simple vaccination card with a real-time photo (a process similar to state-issued identification cards) and anti-counterfeiting technology could be utilized to positively confirm vaccination status without the need to use legal identification. As new variants of the COVID-19 virus become more contagious and more severe for unvaccinated individuals, as evidenced by the Delta variant, the current rise in incidence linked to these variants makes the need for barrier-free vaccination ever more urgent [14]. At this point, controlling the spread of the pandemic is key, and giving individuals greater autonomy and personal responsibility may help increase vaccination rates.

## Abbreviations:

COVID-19: Corona virus disease 2019
IV: Intravenous
OB/GYN: Obstetrician/Gynecologist
